# SOCIAL PARTICIPATION OF PEOPLE LIVING WITH DEMENTIA IN GERMAN SHARED-HOUSING ARRANGEMENTS

**DOI:** 10.1093/geroni/igae098.2044

**Published:** 2024-12-31

**Authors:** Karin Wolf-Ostermann, Julia Misonow, Janissa Altona

**Affiliations:** University of Bremen, Bremen, Bremen, Germany; University of Bremen, Bremen, Bremen, Germany; Institute of Public Health and Nursing Research, Bremen, Bremen, Germany

## Abstract

Aspects of social health are increasingly gaining more interest regarding research for people living with dementia (PlwD). Having a range of meaningful social relationships has been identified as crucial for healthy aging, defined by lower mortality rate, and better physical and mental health outcomes. Recent studies show that social connections and participation in social activities are important dimensions of social health and have the potential to act as a buffer in the process of cognitive decline by delaying dementia or even protecting against its development. Shared-housing arrangements (SHA) in Germany are based on the concept of enabling PlwD to lead an everyday life as normal as possible – including especially social activities as well as exercise-related, sensory, cognitive and creative approaches and out-of-home activities. We analyzed data from different studies on SHA in Germany from 2009-2022 (including the period of the COVID-19 pandemic) to evaluate to which extent social activities are actually performed by PlwD in SHA. The most frequently offered and used activities in SHA are entertainment and joint social activities (2009: 67.0%, 2011: 96.6%, 2021: 80,0%, 2022: 87,9%). This is followed by exercise-related activities (2009: 26.2%, 2011: 79.3%, 2021: 73,2%, 2022: 77,8%) and sensory-related activities (2009: 21.9%, 2011: 77.6%, 2021: 47,9%, 2022: 47,0%). The results show that participation in social activities is a lived fact in SHA - despite some reduction during the pandemic - and should be taken into account when discussing care arrangements for PlwD and conditions for living well with dementia.

